# Validity and Reliability of a Commercial Force Sensor for the Measurement of Upper Body Strength in Sport Climbing

**DOI:** 10.3389/fspor.2022.838358

**Published:** 2022-07-22

**Authors:** Berit K. Labott, Steffen Held, Tim Wiedenmann, Ludwig Rappelt, Pamela Wicker, Lars Donath

**Affiliations:** ^1^Institute of Sport Sciences, Otto-von-Guericke University, Magdeburg, Germany; ^2^Department of Intervention Research in Exercise Training, German Sport University Cologne, Cologne, Germany; ^3^Department of Sports Science, Bielefeld University, Bielefeld, Germany

**Keywords:** mountaineering, strength training, finger flexor muscles, performance, maximal strength

## Abstract

Recreational and professional climbing is gaining popularity. Thus, valid and reliable infield strength monitoring and testing devices are required. This study aims at assessing the validity as well as within- and between-day reliability of two climbing-specific hanging positions for assessing the maximum force with a new force measurement device. Therefore, 25 experienced male (*n* = 16) and female (*n* = 9) climbers (age: 25.5 ± 4.2 years, height: 176.0 ± 9.9 cm, weight: 69.7 ± 14.5 kg, body composition: 11.8 ± 5.7% body fat, climbing level: 17.5 ± 3.9 International Rock Climbing Research Association scale) were randomly tested with climbing-specific hang board strength tests (one-handed rung pulling and one-handed bent arm lock-off at 90°). The Tindeq, a load cell-based sensor for assessing different force-related variables, was employed together with a force plate (Kistler Quattro Jump) during both conditions. Data analysis revealed excellent validity for assessment with Tindeq: The intra-class correlation coefficient (ICC) was 0.99 (both positions), while the standard error of the measurement (SEM), coefficient of variation (CV), and limits of agreement (LoA) showed low values. Within day reliability for the assessment with Tindeq was excellent: rung pulling showed an ICC of 0.90 and arm lock-off an ICC of 0.98; between-day reliability was excellent as well: rung pulling indicated an ICC of 0.95 and arm lock-off an ICC of 0.98. Other reliability indicators such as SEM, CV, and LoA were low. The Tindeq progressor can be applied for the cross-sectional and longitudinal climbing strength assessment as this device can detect training-induced changes reliably.

## Introduction

Climbing in recreational and elite sports settings has received increased attention within the last decades and the sport has become more professional. These developments are reflected by introducing climbing to the 2020 Olympic Games in Tokyo. Olympic climbing in 2020 combined three disciplines: boulder, lead, and speed (International Olympic Committee, 2016). Nearly 10 years before the Tokyo Olympics, the International Rock Climbing Research Association (IRCRA) was founded in order to improve and better understand climbing sport environments, performance, teaching, and coaching on a scientific basis. These advances included anthropometric assessment of (elite) climbers (Watts et al., 1993) or physical performance testing at different climbing levels (Grant et al., 1996, 2001; Kozin et al., 2020). Some studies also compared athletes of different disciplines (boulder, lead, speed) for strength and endurance parameters (Fanchini et al., 2013; Stien et al., 2019; Levernier et al., 2020). Athletes from ice climbing and rock climbing were also compared for anthropometry, muscular strength, endurance, and flexibility (Vujic et al., 2021). Further studies addressed energy costs of climbing such as blood lactate, heart rate, and oxygen-uptake assessment during indoor (Billat et al., 1995) and outdoor climbing (Booth et al., 1999). One more recent study focused on optimal stress loading for climbers (Hill et al., 2020). Relevant factors for successful climbing (Saul et al., 2019) were mainly introduced to elucidate predictors for climbing abilities (Watts et al., 1993; Mermier et al., 2000). Handgrip strength in general as well as fatigue and recovery of the related muscles in particular have often been subjected to climbing-related research (Watts et al., 1996; Grant et al., 2003; Quaine et al., 2003; Labott et al., 2020; Baláš et al., 2021). Thereby, different kinds of holds and their mechanical load have been analyzed (Fuss and Niegl, 2012; Morenas Martín et al., 2013; Vigouroux et al., 2019) along with upper extremity injury prevention studies (Koukoubis et al., 1995). However, not only (fore) arm muscles (Saul et al., 2019; Rokowski et al., 2021) but grip strength is also considered crucial for successful climbing. The important role of adequate leg strength and proper foot technique as well as adequately developed cardiovascular fitness has been emphasized (Larew and Haibach-Beach, 2017). Specific research tools that are commonly employed in climbing research focusing on muscle contractions and maximum force (lift-off force) are electromyography (EMG) electrodes (Ferrara, 2018; Vigouroux et al., 2019), force plates (Watts and Jensen, 2003; Zimny et al., 2016; Vigouroux et al., 2019; Ferrara et al., 2020), force sensors (Fuss and Niegl, 2009; Donath and Wolf, 2015; Vigouroux et al., 2019; Pandurevic et al., 2020), and self-developed load cells devices (Grant et al., 2003; Macleod et al., 2007; Ferrara, 2018; Michailov et al., 2018). Researchers have also suggested to work with a numeric scale measuring maximal strength in different crimp or holding positions (Baláš et al., 2014) or a pulley system (Torr et al., 2020).

For practical purposes, adequate validity and reliability are the major prerequisite for cross-sectional and longitudinal performance assessment in (elite) climbing. Validity of a test instrument refers commonly to the ability to measure accurately and precisely what the respective system is intended to measure. Quantifying validity is commonly performed by comparing the output of the test instrument with a valid and reliable “gold-standard” instrument. Reliability of a test instrument refers to the accuracy of the measurement, e.g., it should contain consistent values over time (test–retest reliability). A high reliability is characterized by minor differences to the force plate, implying intra-class correlation coefficients (ICC) above 0.90 and coefficients of variation (CV) ≤ 10% (Atkinson and Nevill, 1998). Researchers have assessed the reliability of finger flexor strength in different holding positions using electronic scales (Baláš et al., 2014) as an inexpensive and feasible infield approach. They found an intra-class correlation coefficient [ICC; ICC (2,1) and ICC (2,3)] (McGraw and Wong, 1996) for all tested grip positions for test–retest reliability between 0.88 and 0.97 and for intra-session between 0.88 and 0.94, suggesting that this method is a simple, reliable, and valid tool (Baláš et al., 2014). The test–retest reliability of a self-developed system for a strength and endurance test in climbers revealed an ICC [ICC (3,1)] of 0.88 for maximal strength testing with a 95% limit of agreement of 102N (Michailov et al., 2018). Analyzing the reliability for peak force in maximal finger curls using piezoelectric force sensors showed good test–retest reliability for both hands left: *R* = 0.947 [0.95 confidence interval (*CI*), 0.891–0.975] and right: *R* = 0.902 (0.95 *CI*, 0.796–0.953) (Watts and Jensen, 2003). Using a pulley system for maximal isometric finger strength revealed an ICC (3,1) for test–retest reliability >0.91 (Torr et al., 2020).

A newly developed device for analyzing climbing-specific force parameters in a real-life setting is the “progressor” of Tindeq (Tindeq, Trondheim, Norway) which is a load cell-based sensor for assessing different force-related variables (Tindeq) (Tindeq Progressor, 0000). This device could make climbing-related assessments and research more feasible and easily applicable for coaches and athletes. Although the device cannot be mounted directly onto the climbing wall, the Tindeq can be used in free-hanging positions. Regarding ecological validity, which describes the generalization of findings from the lab to the ‘in field' or actual situation of an athlete's training or competition (Kihlstrom, 2021), it could be used to analyze quick and easy forces during different free-hanging tasks in the gym or outdoors.

For accurate monitoring of an athlete's training process and progression, it is of utmost importance that relevant measures can be obtained reliably and validly (Atkinson and Nevill, 1998; Hopkins et al., 2001; Hopkins, 2004). Tools and tests of interest need to be able to detect small changes and differences in an athlete's performance surrogate (Atkinson and Nevill, 1998; Hopkins, 2004) precisely. Thus, relative reliability components of measurement error need to be addressed (Atkinson and Nevill, 1998).

Against this background, the aim of this study was to compare maximum force indicators during two hanging positions with the training and monitoring device from Tindeq with a force plate in terms of validity and reliability. This was done by recording the maximum force of climbers performing different established climbing-specific hang board strength tests. Absolute and relative reliability parameters of both devices were assessed. The researchers' hypothesis was that the assessment with the Tindeq progressor serves as an excellently valid and reliable measurement device assessing maximum force during static hanging positions compared to measurements with a force plate.

## Materials and Methods

### Participants

Included participants were 25 experienced male and female climbers [>6a of Union Internationale des Associations d' Alpinisme scale (International Mountaineering and Climbing Federation)] at the age of 18–40 years. Participants were excluded (a) when they experienced shortness of breath, chest discomfort during daily activities, and difficulty, pain, or dizziness when climbing stairs, (b) when they had recent serious cardiovascular events (e.g., heart failure or uncontrolled hypertension), (c) when they had acute infections or other diseases, or (d) when physical limitations that prevent intensive training (e.g., severe orthopedic diseases, especially of the upper extremity) were present. Data collection included age, height, weight, activities, and body composition which was assessed using a bioimpedance analysis (BIA) to determine body fat percentage. Characteristics of the included participants are presented in Table 1. All participants were familiar with the applied test protocol.

**Table 1:** Participant characteristics, means with standard deviations (SD).

**Characteristic**	**Female (*n* = 9)**	**Male (*n* = 16)**	**All (*N* = 25)**
Age (years)	25.2 (5.3)	25.6 (3.6)	25.5 (4.2)
Height (cm)	167.0 (6.5)	181.1 (7.6)	176.0 (9.9)
Weight (kg)	58.4 (9.3)	76.4 (12.8)	69.7 (14.5)
Body Fat (%)	15.2 (6.8)	9.8 (3.8)	11.8 (5.7)
IRCRA rating scale (a.u.)	16.2 (3.8)	18.2 (3.8)	17.5 (3.9)

*IRCRA, International Rock Climbing Research Association*.

The present study has been designed as a validation and reliability study with two points of measurement. The study was conducted at the German Sport University Cologne (Cologne, Germany) and was approved by the ethical committee (ethical approval number: 064/2021). All participants signed an informed consent and the study design and conduction complied with the Declaration of Helsinki.

### Study Design

Each participant took part in a total of two sessions (~120 min each). After receiving all relevant study information and a standardized warm-up (described in detail below), four climbing-specific strength tests (free one-handed rung pulling, fixed one-handed rung pulling, free one-handed bent arm lock-off at 90°, and fixed one-handed bent arm lock-off at 90°) were performed. All participants initially performed the one-handed rung pulling and then the one-handed bent arm lock-off. The order of testing conditions (free or fixed) was randomized, but was the same for each participant at both test appointments. During the ‘free' conditions, the Tindeq progressor (Tindeq, Trondheim Norway) was used for recording in addition to the force plate, during the ‘fixed' conditions only the force plate (Quattro Jump, Kistler, Winterthur, Switzerland) recorded the data.

Prior to maximum force testing, participants performed a general and specific warm-up. The general warm-up consisted of individual mobility exercises and light dynamic pulling with resistance ribbons for 2 min. This was followed by the specific warm-up, which consisted of three or four submaximal warm-up sets for the 90° lock-off (arm) and the crimp (finger), respectively. The specific warm-up was performed on the installed equipment and was designed to mimic the exact muscle action of the tests. The load:rest ratio was 3:3 s in each set. In between the sets, the rest was 60 s. Participants performed four warm-up sets. They were instructed to reach a predetermined intensity (rate of perceived exertion (RPE)) measured *via* a 10-scaled Borg-Scale (Foster et al., 2001). In the first warm-up set, the RPE should be 4, in the second and third 6, and in the last set 8. The amount of repetitions in sets 1–4 was 8, 6, 6, and 4, respectively. Afterwards, three repetitions of maximal force testing were performed. The whole test battery consisted of three maximal repetitions for each of the four tests starting with the one-handed rung pulling. The rest period between the three repetitions was 90 s. The testing battery was conducted two times per appointment with a 15 min break in between. In total, all participants performed 24 maximal muscle contractions (2 test batteries × 4 tests × 3 maximal repetitions) per lab visit. The maximal force of the finger flexor muscles (free one-handed rung pull) was measured for the dominant hand, while the static maximal force of the upper body traction muscles (one-handed bent arm lock-off at 90°) was assessed for the non-dominant side. Both devices were calibrated prior to each measurement as specified by the manufacturer.

The maximum force of the finger flexor muscles was measured using the ‘one-handed rung pulling' (Augste et al., 2020): Participants stood on a force plate and tried to pull themselves off the ground holding one-handed on a fingerboard with a 20 mm bar, which was installed above head height. This test was performed under two conditions: free and fixed. During the free condition, the fingerboard was free-hanging from the ceiling; it was not static but movable. During the fixed condition, it was attached to a rigid construction, here it was attached to a hanging board which is commonly used. The Tindeq progressor was used in the free condition and was placed between the hanging structure from the ceiling and the fingerboard. The free condition was applied as the Tindeq sensor cannot be attached to a vertical wall, it can only be used in a free-hanging condition. Both test conditions followed the same test protocol and were conducted in the same manner. Participants hanged/hung with a crimp grip on the bar, and then they performed a light squat so that the elbow is extended and the center of gravity was perpendicular under the bar. On the command of the test assessor, participants pulled the bar for 3 s, thereby relieving the force plate. As in climbing, the load of the finger flexor muscles began with a short eccentric phase before the static position was reached. The maximum force of the finger flexor muscles was determined from the difference in weight force. When a participant was able to pull himself completely up, additional weights were added to the waist belt.

The static maximum force of the upper body traction muscles was measured using the ‘one-handed bent arm lock-off at 90°' (Augste et al., 2020): Participants stood on a force plate and tried to pull themselves off the ground holding with one arm a pull-up bar which was installed in head height. This test was performed under two conditions: free and fixed. In the free condition, the pull-up bar was free-hanging and moveable. During the fixed condition, the bar was attached to a rigid construction in order to be static. The Tindeq progressor was also used in the free condition and was hanging in between the handle and the hanging construction from the ceiling. Participants grabbed the pull-up bar with an elbow angle of 90°, and on command of the test assessor, participants pulled the bar for 3 s, thereby relieving the force plate. The maximum force of the upper traction muscles was determined from the difference in weight force. When a participant was able to pull himself completely up, additional weights were added to the waist belt.

All trials were recorded on the force plate at a sampling rate of 500 Hz. Further, all trials of the ‘free' condition were simultaneously recorded by the Tindeq progressor at a sampling rate of 80 Hz and transferred *via* Bluetooth® to a mobile phone. The moving average over a time frame of 3 s was calculated for each trial (Augste et al., 2020). Subsequently, for the force plate, the difference between the participants' weight (determined by the force applied to the force plate prior to each trial) and the minimum value of the force-time-curve was calculated. For the Tindeq progressor, the maximum value of the force–time curve was determined and divided by *g* = 9.81 m/s^2^ in order to convert the collected data from kg into N. For both devices, the best two trials (i.e., the largest force applied) out of three were averaged and used for all further analyses.

### Statistical Analysis

All data are presented as means with standard deviations (SD) or with 95% *CI*s. All measured outcomes were checked for normal distribution using Shapiro–Wilk test and for variance homogeneity using the Levene test and found to be non-significant (*p* > 0.05). The statistical significance was set at an α-level of 0.05. A one-factorial repeated measurement analysis of variance (rANOVA) was conducted for testing positions in the free condition (device: Tindeq *vs*. Kistler) and for comparing the two conditions (free *vs*. fix) in testing positions measured with the Kistler force plate. Several one-factorial rANOVAs were computed for between and within day reliability (within day: test 1 *vs*. test 2; between day: day 1 *vs*. day 2) for all conditions measured with the Tindeq Progressor. The rANOVA effect sizes are presented as $\eta_{\text{p}}^{2}$ with values ≥0.01 indicating small, ≥0.06 moderate, and ≥0.14 indicating large effects (Cohen, 1988). Standardized mean differences (SMD) were calculated as the difference between both conditions divided by the pooled SD of both conditions (Atkinson and Nevill, 1998) as a measure of the pairwise effect size estimation. SMD values are interpreted as trivial: < |0.2 |, small: |0.2 | ≤ SMD < |0.5 |, moderate: |0.5 | ≤ SMD < |0.8 |, and large SMD: ≥ |0.8 | (Cohen, 1988). For analyzing, the agreement of both devices and between- and within-day reliability, limits of agreement (LoA) were calculated with 1.96^*^SD, considering a 95% random error component (Atkinson and Nevill, 1998), and Bland–Altman plots were created (Martin Bland and Altman, 1986). Furthermore, standard error of measurement [SEM; SEM = SD$\sqrt{\left(1--ICC\right)}$], coefficient of variation (CV; CV = SD/mean ^*^ 100), and ICCs were calculated (Atkinson and Nevill, 1998). ICCs were calculated as random effect variance divided by the total variance, i.e., the sum of the random effect variance and the residual variance. Depending on the obtained value, the ICC was considered as excellent (0.9 to 1), good (0.74 to 0.9), moderate (0.4 to 0.73), and poor (0 to 0.39) (Fleiss, 1999). Statistical analyses were conducted using R (version 4.0.5) and RStudio (version 1.4.1106) software.

## Results

### Validity Tindeq

**Figure 1:**
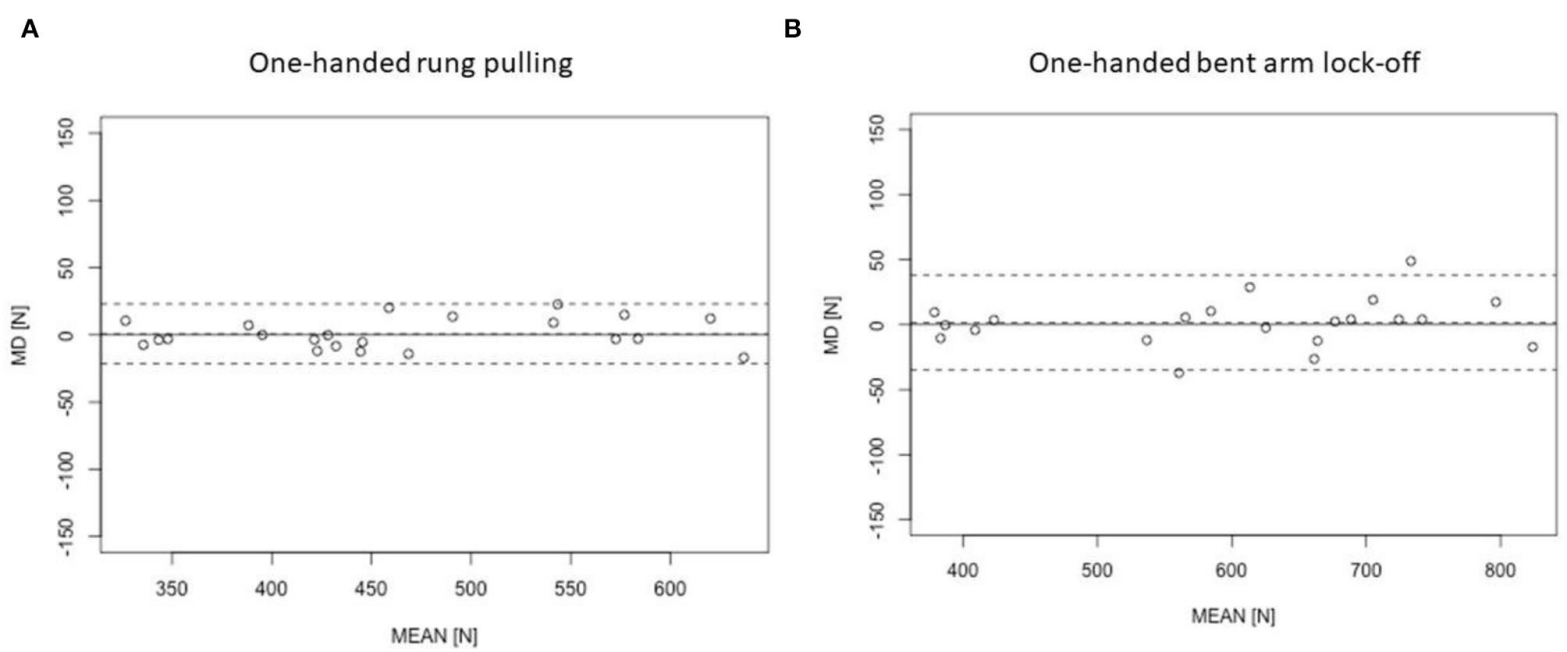
Bland–Altman plots (MD, mean difference between both devices; MEAN, average of both devices) for the validity of the Tindeq *vs*. Kistler for **(A)** one-handed rung pulling and; **(B)** one-handed bent arm lock-off. The dashed line indicates 95% limit of agreement. The first test session is displayed.

The rANOVA showed no statistically significant difference between both devices for the one-handed bent arm lock-off (*p* = 0.687, $\eta_{p}^{2}$ = 0.008 (95% *CI* 0; 0.20), *SMD* = 0.011) and for the one-handed rung pulling (*p* = 0.767, $\eta_{p}^{2}$ = 0.004 (95% *CI* 0; 0.17), *SMD* = 0.008). ICCs, CV, SEM, and LoA values (Figure 1) indicate the measures of validity between both devices in the free conditions (Table 2).

**Table 2:** Validity and reliability indicators for the comparison between Tindeq *vs*. Kistler in the free condition.

**Test**	**Device**	**Mean (SD) [*N*]**	**MD (SD) [*N*]**	**SEM [*N*]**	**CV [%]**	**ICC (95% CI)**	**LoA [*N*]**
One-handed rung pulling	Kistler	465 (95)	1 (11)	0.9	2.4	0.99 (0.98; 1.00)	22
	Tindeq	464 (94)					
One-handed bent arm lock-off	Kistler	604 (142)	2 (19)	1.7	3.1	0.99 (0.98; 1.00)	37
	Tindeq	603 (139)					

*MD, Mean Difference; SD, Standard Deviation; SEM, Standard Error of Measurement; CV, Coefficient of Variation; ICC, Interclass Correlation Coefficient; CI, Confidence Interval; LoA, Limits of Agreement*.

### Validity Testing Positions Kistler

**Figure 2:**
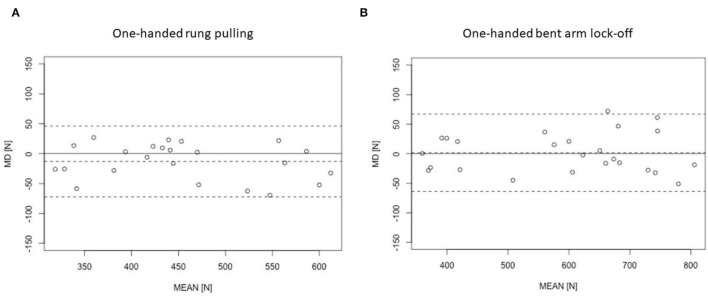
The Bland–Altman plots [MD, mean difference between both testing conditions (free and fixed); MEAN, average of both conditions] for the validity of the testing conditions measured *via* Kistler force plate for **(A)** one-handed rung pulling and; **(B)** one-handed bent arm lock-off. The dashed line indicates 95% limit of agreement. The first test session is displayed.

A comparison of both conditions (free *vs*. fixed) yielded the following results: For the one-handed rung pulling, a statistically significant difference (*p* = 0.049, ηp^2^ = 0.165 (95% *CI* 0; 0.41), *SMD* = −0.148) was found, but not for the one-handed bent arm lock-off [*p* = 0.800, ηp^2^ = 0.003 (95% *CI* 0; 0.14)]. Differences between free and fixed one-handed rung pulling were analyzed *via* SMD = 0.012. Both, the one-handed bent arm lock-off and the one-handed rung pulling (Table 3) revealed high ICCs and low CV, SEM, and LoA (Figure 2), representing measures of validity between the fixed and the free condition for the one-handed bent arm lock-off and the one-handed rung pulling.

**Table 3:** Comparison of the two conditions (free *vs*. fixed): validity and reliability indicators for both testing positions, measurements recorded by the Kistler force plate.

**Test**	**Condition**	**Mean (SD) [*N*]**	**MD (SD) [*N*]**	**SEM [*N*]**	**CV [%]**	**ICC (95% CI)**	**LoA [*N*]**
One-handed rung pulling	fix	447 (89)	−13 (30)	7.5	6.6	0.94 (0.85; 0.97)	59
	free	461 (95)					
One-handed bent arm lock-off	fix	591 (146)	2 (33)	5.3	5.7	0.97 (0.94; 0.99)	66
	free	590 (145)					

*MD, Mean Difference; SD, Standard Deviation; SEM, Standard Error of Measurement; CV, Coefficient of Variation; ICC, Interclass Correlation Coefficient; CI, Confidence Interval; LoA, Limits of Agreement*.

### Within and Between Day Reliability Tindeq

The rANOVA revealed no significant differences for within day (*p* = 0.082, ηp^2^ = 0.167 (95% *CI* 0; 0.44, *SMD* = 0.181) and between day (*p* = 0.252, ηp^2^ = 0.060 (95% *CI* 0; 0.30), *SMD* = −0.077) one-hand rung pulling and for between day (*p* = 0.975, ηp^2^ > 0.001 (95% *CI* 0; 0), *SMD* = −0.001) one-handed bent arm lock-off reliability of the Tindeq sensor. Only the within day, reliability of the one-handed bent arm lock-off revealed a significant rANOVA effect (*p* < 0.001, ηp^2^ = 0.554 (95% *CI* 0.21; 0.72). The pairwise effect size between days during within day reliability of the one-handed bent arm lock-off was found to be *SMD* = 0.163. Within and between day reliability for both positions is represented by measures of SEM and CV as well as ICC values of the Tindeq sensor (Table 4). The LoA (<71N) values further support this (Table 4).

**Table 4:** Within-day and between-day reliability indicators for Tindeq in the free-hanging position.

		**Mean (SD) [*N*]**	**MD (SD) [*N*]**	**SEM [*N*]**	**CV [%]**	**ICC (95% CI)**	**LoA [*N*]**
Within day reliability (one-handed rung pulling)	Test 1	452 (88)	16 (36)	11.2	8.2	0.90 (0.76; 0.96)	71
	Test 2	436 (89)					
Within day reliability (one-handed bent arm lock-off)	Test 1	603 (142)	23 (21)	3.3	3.6	0.98 (0.75; 1.00)	42
	Test 2	580 (137)					
Between day reliability (one-handed rung pulling)	Day 1	464 (94)	−7 (29)	6.6	6.2	0.95 (0.88; 0.98)	57
	Day 2	472 (86)					
Between day reliability (one-handed bent arm lock-off)	Day 1	603 (142)	0 (27)	3.4	4.4	0.98 (0.96; 1.00)	52
	Day 2	603 (144)					

*MD, Mean Difference; SD, Standard Deviation; SEM, Standard Error of Measurement; CV, Coefficient of Variation; ICC, Interclass Correlation Coefficient; CI, Confidence Interval; LoA, Limits of Agreement*.

## Discussion

This study is the first that assessed the validity and (within and between day) reliability of the new force measuring device by Tindeq on two different climbing-related holds (one-handed rung pull up and one-handed bent arm lock-off) in experienced climbers. Based on the low standard error of measurement and coefficient of variation (<10%), the high interclass correlation coefficient (≥0.90), and the small limits of agreements, the assessment using Tindeq can be considered a valid and reliable (within and between day) measurement instrument for the here applied testing positions.

The present results showed low CV scores (≤8.2%) for between and within day reliability of Tindeq. The limits of agreement, which estimate the interval (95%) including a proportion of the differences between measurements (Giavarina, 2015), also indicate the validity of Tindeq with measurements between 22 and 71N. Comparing the assessments with Tindeq and the force plate using CV, SEM, or ICC as indicators of validity (the agreement of both devices), the findings suggest an excellent agreement.

Other devices for measuring climbing-related forces have been tested for validity and reliability before. Reliability was assessed for an electronic scale for measuring finger flexor strength measurement in different grip positions (Baláš et al., 2014). Grip positions that were applied on a climbing hold were open grip, crimp grip, index and middle finger, and middle and ring finger. Data were recorded at a recording rate of 20 Hz. The presented ICC values for all grip positions ranged between 0.88 and 0.97 for within day reliability and between 0.88 and 0.94 for between day reliability. The authors reported just these reliability parameters (within and between days). Compared to the present ICC values, those of the electronic scale are slightly lower. Thus, the assessment with the Tindeq is superior to the scale with regard to within and between day reliability. The testing position was similar to the one in the present study: participants stood on a scale and tried to pull themselves off the scale in different holding positions. They held onto a wooden finger board attached to a wall. However, participants tried to pull themselves up on a free hanging board or handle in the present study. The free-hanging set-up may not reflect the conditions during climbing on a rock or wall. Nevertheless, the comparison of the free-hanging and the fixed condition shows that the conditions are in accordance with each other.

Other authors included force sensors in the crimps on the climbing wall to measure the maximum resultant force during bouldering a route with different wall inclinations (Donath and Wolf, 2015). Mean forces in different climbing crimps were found to have different within day reliability values (ranging from 0.63 to 0.93) with a CV between 5 and 10%. The assessment with the sensors in the underclining crimp grip received the best values regarding reliability (95% CI (0.8;1.0), CV 5.8%, SEM 1.7) and validity (paired Student's *t*-test *p* = 0.55). Compared to the present results of the assessment with Tindeq, the integrated force sensors have partly lower reliability values (ICC), while the CV of the integrated sensors is good. The integrated sensors can be regarded as both—advantageous due to the standardized situation indoors and disadvantageous for quick and easy assessments somewhere else. Different grips could also be applied with the Tindeq, but no fixed conditions can be simulated while using the new sensor. Furthermore, a lot of technical equipment is necessary to record data of the integrated sensors on the climbing holds.

Regarding maximal finger force or endurance assessment, different approaches exist. A reliable assessment for finger force measurements in climbers was proposed by Watts and Jensen (Watts and Jensen, 2003), who reported excellent test-retest reliability values [right hand 0.90 (95% *CI* 0.79–0.95), left hand 0.94 (95% *CI* 0.89–0.97)]. Despite these promising values, the testing position was standardized, but it was not highly climbing-specific as the participants were seated in front of the testing device and performed a 3 s maximal contraction. The testing device used in the study led by Watts and Jensen included a piezoelectric force sensor fitted with a rigid plate and recorded at a sample frequency of 500 Hz. Another group of researchers analyzed the impact of arm-fixation on maximal finger strength and endurance (Michailov et al., 2018) with a self-developed device (recording sample rate 125 Hz), i.e., a three-dimensional (3D) system for the performance assessment in rock climbing (3DSAC). They used the fixation to create a standardized situation in which other muscles (shoulder girdle muscles) did not enhance the fingertip force production. However, the researchers were aware that this situation could lead to a more standardized testing procedure, while reflecting a less climbing-specific position. The ICC for test-retest reliability assessment without fixation of the 3DSAC (Michailov et al., 2018) stated as at 0.88 with a 95% LoA of 102N. Regarding ICC values for the condition with fixation, higher values were reported (ICC 0.94) and the 95% LoA was also lower (75N). In the present study, the ICC of the assessment with Tindeq ranged between 0.91 and 0.99 and the LoA was between 22N and 71N, collectively indicating better reliability than the 3DSAC. However, involved muscle groups and forces were slightly different (fingertip *vs*. finger flexors/upper body traction muscles). A recently published study analyzed the most reliable depth of the fingerboard for maximum hanging time of climbers (López-Rivera et al., 2022). Different depths (6, 8, 10, 12, and 14 mm) were tested. Participants performed dead hangs until muscular failure, while hanging time was recorded. Although not all climbers were able to hold onto the 8 mm bar, the authors suggested this depth for climbers of level 6b-8c of the IRCRA scale as the most reliable depth for maximum hanging time. In the present study, however, participants had a depth of 20 mm for the one-handed rung pulling test and a standardized width of the bar during the bent-arm lock-off.

The employed Tindeq progressor is a commercially available product. The manufacturer is only providing limited information on the technical details, configurations, settings, and data filtering. The data (as maximum force and rate of force development) can be inspected *via* a smartphone application. The essential data are, however, visible for coaches and athletes. During daily training and performance testing, the different variables could be useful, depending on the athlete's training goals (increase maximum force, endurance, or rapid force application). A valid measurement of the force parameters in hanging or pulling tasks helps assessing training progress and can build a base for monitoring the progress of the strength training. Measuring the variables with different methods, the LoA should be taken in account by a coach in order to see if the different devices are indicating likewise results. The limits of agreement is a range where 95% of all values always lie within. It includes systematic (bias) and random error (precision), and provides a useful measure for comparing the likely differences between individual results measured by two methods (Giavarina, 2015). Limits of agreement of 20–30N would be able to detect changes of 2–3 kg in the athlete's performance (≤5% of the performance of the athletes in the present study). In elite climbers, an increase in grip strength of 5–8% has been reported after 4 weeks of grip training (Levernier and Laffaye, 2019). Further, after 4 weeks of training dead hangs, an increase of >9% in grip strength was reported for experienced climbers (López-Rivera and González-Badillo, 2012). Therefore, these LoAs seem to be low enough for detecting expected changes in both sub-elite and elite climbers and thus be useful for coaches or athletes. During this study, we did not assess fatigue but every participant was his or her own control group. The Tindeq progressor is attached to a handle/board and scaffold or ceiling and can be used in- and outdoors for measuring diverse parameters. The sensor is not attached *via* a cable for data processing, but transmits data *via* Bluetooth and an application to a smartphone. Climbers and coaches looking for a feasible solution in determining strength can use this device for reliable and valid testing.

Nevertheless, some limitations of this study need to be addressed. All participants were experienced climbers. Further research should examine if these maximum strength measurements are also reliable in less experienced climbers who may have less strong upper extremity muscles. Therefore, participants' climbing level should be carefully assessed. It is of importance to highlight that only static hanging positions were assessed during this study. However, these established static positions are of high relevance in climbing diagnostic. Further, applying the moving average method over a period of 3 s to determine the minimum (force plate) and maximum force (Tindeq progressor) and subsequently averaging the two best trials most likely leads to a smaller difference between the two test approaches and, therefore, a higher reliability of the test. However, we decided to use this time frame of 3 s, as both the ‘one-handed rung pulling' test and the ‘bent arm lock-off' are evaluated by averaging the applied force on the force plate over a time frame of 3 s (Augste et al., 2020).

## Conclusions

The Tindeq progressor was found to be a highly valid and reliable measure instrument for maximal forces using the present assessments. When performing one-handed rung pull-ups and one-handed bent arm lock-offs, it showed good to excellent validity and (within and between day) reliability with low limits of agreements for the mean maximum force during both positions. Thus, the commercially available Tindeq progressor can be considered a feasible and applicable tool for the detection training-induced changes in climbing-related strength measures in non-lab settings.

## Data Availability Statement

The raw data supporting the conclusions of this article will be made available by the authors, without undue reservation.

## Ethics Statement

The studies involving human participants were reviewed and approved by Ethics Committee of the German Sport University Cologne. The patients/participants provided their written informed consent to participate in this study.

## Author Contributions

Conceptualization, methodology, and funding acquisition: LD and SH. Software, validation, formal analysis, and visualization: SH. Investigation and data curation: TW and LR. Writing—original draft preparation: BL. Writing—review and editing: SH, PW, and LD. Supervision and project administration: LD. All authors have read and agreed to the published version of the manuscript.

## Funding

This research was funded by the German Federal Institute of Sport science (BISp), grant number 071602/21. We also acknowledge the financial support of the German Research Foundation (DFG) and the Open Access Publication Fund of Bielefeld University for the article processing charge.

## Conflict of Interest

The authors declare that the research was conducted in the absence of any commercial or financial relationships that could be construed as a potential conflict of interest.

## Publisher's Note

All claims expressed in this article are solely those of the authors and do not necessarily represent those of their affiliated organizations, or those of the publisher, the editors and the reviewers. Any product that may be evaluated in this article, or claim that may be made by its manufacturer, is not guaranteed or endorsed by the publisher.
